# Supplementary material for: Modulation of signature cancer-related genes in oral cancer cells (Ca9-22) by anethole treatment: Insights into therapeutic potential
Source: PLoS One. 2024 Dec 17;19(12):e0315085. doi: 10.1371/journal.pone.0315085 (PMC11651572; doi:10.1371/journal.pone.0315085)
Supplement: Attachment — Submitted filename: Response to reviewer coments ( PlosOne).docx [file pone.0315085.s001.docx]

**Journal Requirements:**

1. When submitting your revision, we need you to address these additional requirements.

Please ensure that your manuscript meets PLOS ONE's style requirements, including those for file naming. The PLOS ONE style templates can be found at 
<https://journals.plos.org/plosone/s/file?id=wjVg/PLOSOne_formatting_sample_main_body.pdf> and 
<https://journals.plos.org/plosone/s/file?id=ba62/PLOSOne_formatting_sample_title_authors_affiliations.pdf>

**Response.** All it done

2. Thank you for stating the following financial disclosure: 
Deanship of Scientific Research at Imam University (IMSIU) (grant number IMSIU-RP23099).
  
Please state what role the funders took in the study.  If the funders had no role, please state: "The funders had no role in study design, data collection and analysis, decision to publish, or preparation of the manuscript." 
If this statement is not correct you must amend it as needed. 
Please include this amended Role of Funder statement in your cover letter; we will change the online submission form on your behalf.

**Response**. We confirm that. The funders had no role in study design, data collection and analysis, decision to publish, or preparation of the manuscript.

3. Thank you for stating the following in the Acknowledgments Section of your manuscript: 
The authors extend their appreciation to the Deanship of Scientific Research at Imam University (IMSIU) (grant number IMSIU-RP23099).

We note that you have provided funding information that is not currently declared in your Funding Statement. However, funding information should not appear in the Acknowledgments section or other areas of your manuscript. We will only publish funding information present in the Funding Statement section of the online submission form. 
Please remove any funding-related text from the manuscript and let us know how you would like to update your Funding Statement. Currently, your Funding Statement reads as follows: 
Deanship of Scientific Research at Imam University (IMSIU) (grant number IMSIU-RP23099).

Please include your amended statements within your cover letter; we will change the online submission form on your behalf.

**Response**. We have added the sentence (This work was supported and funded by the Deanship of Scientific Research at Imam Mohammad Ibn Saud Islamic University (IMSIU) (grant number IMSIU-RP23099) in funding section

4. We note that your Data Availability Statement is currently as follows: All relevant data are within the manuscript and its Supporting Information files.

Please confirm at this time whether or not your submission contains all raw data required to replicate the results of your study. Authors must share the “minimal data set” for their submission. PLOS defines the minimal data set to consist of the data required to replicate all study findings reported in the article, as well as related metadata and methods (<https://journals.plos.org/plosone/s/data-availability#loc-minimal-data-set-definition>).

For example, authors should submit the following data:

- The values behind the means, standard deviations and other measures reported;
- The values used to build graphs;
- The points extracted from images for analysis.

**Response .** We confirm that all raw data required to replicate the results were in the manuscript

**Additional Editor Comments:**

Please consider the reviewer's comments. I ask for special attention to text corrections related to English.

**Response. It done**

**Reviewer #1:** The manuscript describes the effect of Anethole treatment on oral cancer cells (Ca9-22) using in vitro functional assays and bioinformatics. The authors examined the impact of Anethole on cell proliferation, cell cycle, and apoptosis. Moreover, PCR array analysis was employed to investigate the alterations induced by Anethole’s treatment in the overall expression of genes associated with the cell cycle, apoptosis, oncogenes, and tumor suppressor genes. Overall, the data are convincing, and those on cell proliferation and apoptosis are not new. However, the novelty of the paper resides in the PCR array and the related analysis by bioinformatics of the different genes and proteins and their interactions. From my point of view, the study lacks validation of such PCR array/bioinformatics analysis, at least for one or two key gene/protein interactions. Finally, the results section needs improvement in terms for format and style and the whole manuscript needs substantial English/grammatical checks and revision.

Here are my major comments:

- The abstract is too long and needs reshaping.

**Response**. The abstract was changed according to your nice suggestion (less than 350 words)

- The manuscript’s text presents many formatting issues that need to be fixed either by the authors or the journal.

**Response**. Thank you for this comment. The manuscript has been formatted according to the instructions of plos one

- Overall, the manuscript presents many typos and unclear sentences. This needs substantial English/grammatical checks and revision.

**Response.** A global revision of the manuscript was carried out by a company specializing in English editing to make it easy to read and understand**.**

- Everywhere, “Cell cycle” needs to be changed to “cell cycle”.

**Response.** Thank you for your remark. It done

- Line 161, the section related to RNA extraction by TRIzol reagent, must be moved one line down in a separate paragraph.

**Response.** Sorry for this error. It done.

- Figure 1A/B, no need to show the structure of Anethole.

**Response. I agree.** The structure was removed from figure 1 as suggested.

- The results section related to Figure 1 contains many % and p values. This could be summarized in a table and keep the section as text-based descriptive.

**Response.** As suggested, Table 1 was added in section 1 of results.

- Figure 1C is a bit confusing with different count scales (Y-axis). Visually, one would interpret that Anethole treatment increased both G0/G1 and S/G2/M population. Therefore, if both graphs had the same Y-axis scale set up to 350, G0/G1 curve should indeed be lower with Anethole treatment, BUT I AM NOT SURE for S/G2/M curves ??? It looks like S phase-curve is around 50 counts in both conditions. The authors need to clarify this and re-consider their conclusions from this experiment.

**Response.** Yes the figure is correct and we have added the percentages of each population in each figure to avoid confusion

- The sections on Cell cycle RT2 233 Profiler PCR Arrays for both cell cycle (Figure 2) and apoptosis (Figure 5) are very dense and very difficult to read with all the genes and their respective folds of change values. All this needs to be summarized in a separate table that would be related to Figures 2 and 5.

**Response.** Thank you for your nice suggestion. The figure 2 and fig 5 were changed and we have introduced a new table to simplify the understanding of data

- The study lacks afunctional validation of some key genes/proteins identified potentially modulated by Anethole from Cell cycle RT2 233 Profiler PCR Arrays. Thus, since only 1 gene (the cyclin-dependent kinase inhibitor 1A) for cell cycle and 2 genes (V-raf-1 murine leukemia viral 364 oncogene homolog 1(RAF1) and retinoic acid receptor, alpha (RARA)) for apoptosis were upregulated by Anethole, I strongly ask to perform a basic validation experiment in Ca9-22 oral cancer cells where the authors may either silent these genes using siRNA or quantify their levels by western blot. Such a validation will provide an added value to the study.

**Response.** We have already anticipated this question and we have added a figure 7 shown the western blotting analysis for the expression of 4 key genes involved in cell cycle and apoptosis. In next study we will investigate the role of miRNA on expression of theses genes and also to stydy their profile of methylation. Thank you for your nice suggestion.

- I found it surprising that none of the genes modulated by Anethole’s treatment coded for caspases, despite its demonstrated apoptotic action! Any explanation or speculation from the authors?

**Response.** I agree your suggestion, In figure 7 we have added the expression of Caspase 8 down regulated by anethole

- In the discussion and conclusion, it’s surprising that the authors did not focus on the 3 genes (the cyclin-dependent kinase inhibitor 1A, for cell cycle, and V-raf-1 murine leukemia viral 364 oncogene homolog 1/retinoic acid receptor, for apoptosis) were upregulated by Anethole treatment. The upregulation of these genes may rationally explain the anti-cancer effects of Anethole.

**Response.** The conclusion section was changed as suggested by reviewers . Also results section and figures according to new analysis by normalizing by two housekeeping genes
